# Nogo-B promotes the epithelial-mesenchymal transition in HeLa cervical cancer cells via Fibulin-5

**DOI:** 10.3892/or.2022.8311

**Published:** 2022-03-28

**Authors:** Wei Xiao, Shumin Zhou, Hua Xu, Heng Li, Guoqing He, Yingle Liu, Yipeng Qi

Oncol Rep 29: 109–116, 2013; DOI: 10.3892/or.2012.2069

Following the publication of the above article, an interested reader drew to the authors’ attention that the ‘NB-4’ and ‘NB-2’ panels for the invasion and migration assays shown in Fig. 3B and C on p. 113 appeared to contain overlapping data, such that the data may have been derived from the same original source, even though the panels were intending to have shown results obtained under different experimental conditions. The authors have re-examined their raw data and realized that these data were inadvertently mixed up when Fig. 3B and C were assembled.

A corrected version of Fig. 3, showing the data as they should have appeared for the ‘NB-4’ and ‘NB-2’ invasion and migration assay experiments in Fig. 3B and C, is shown on the next page. The authors sincerely apologize for the errors that were introduced into Fig. 3 of the published article, and thank the Editor of *Oncology Reports* for allowing them the opportunity to publish a Corrigendum. All the authors agree to the publication of the authors, and they apologize to the readership for any inconvenience caused.

## Figures and Tables

**Figure 3.**
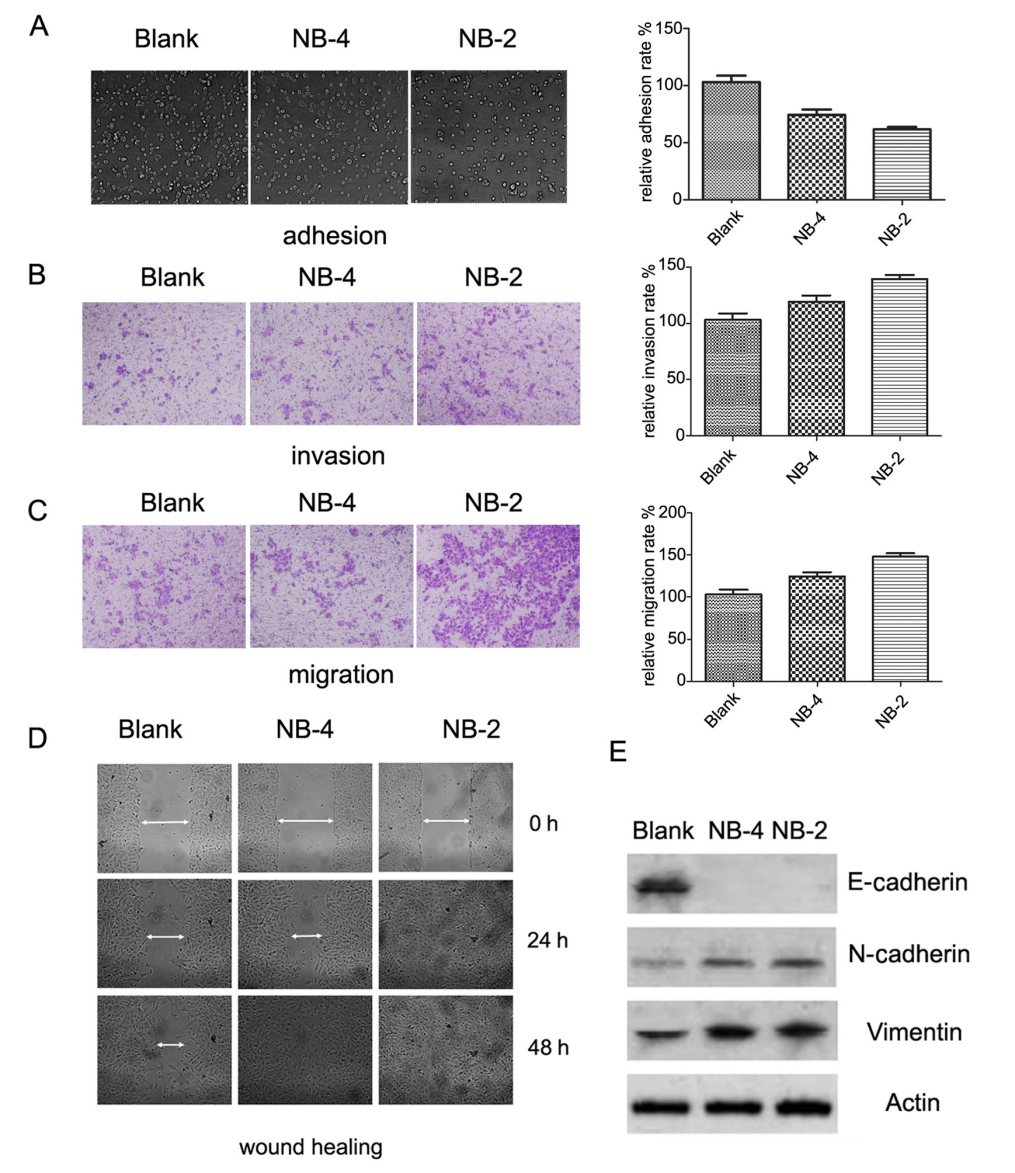
Nogo-B promotes EMT in HeLa cells. The adhesion (A), migration (B) and invasion (C) of NB-2, NB-4 and Blank cells were examined by MTT or transwell assays. The left images show the experimental results from one experiment, while the right histogram shows three independent assays presented as the means ± SE. The data are presented as percentages relative to Blank cells. (D) The *in vitro* wound healing assay exhibited different migration abilities of the NB-2, NB-4 and Blank cells. Additionally, the NB-2 and NB-4 cells also exhibited a random migration pattern, which has been described as a novel mesenchymal behavior. (E) Immunoblot analysis of epithelial (E-cadherin) and mesenchymal (N-cadherin and vimentin) marker proteins in NB-2, NB-4 and Blank cells.

